# Efficacy of Engraftment and Safety of Human Umbilical Di-Chimeric Cell (HUDC) Therapy after Systemic Intraosseous Administration in an Experimental Model

**DOI:** 10.3390/biomedicines12051064

**Published:** 2024-05-11

**Authors:** Maria Siemionow, Lucile Chambily, Sonia Brodowska

**Affiliations:** 1 Department of Orthopaedics, University of Illinois at Chicago, Chicago, IL 60607, USA; lucile.chambily@gmail.com (L.C.); soniaa.bro@gmail.com (S.B.); 2 Department of Traumatology, Orthopaedics, and Surgery of the Hand, Poznan University of Medical Sciences, 61-701 Poznan, Poland

**Keywords:** biodistribution, cell-based therapy, donor-specific chimerism, human umbilical di-chimeric cell (HUDC) therapy, regenerative medicine, safety, systemic intraosseous administration, tolerance induction, transplantation, umbilical cord blood (UCB) cells

## Abstract

Cell-based therapies hold promise for novel therapeutic strategies in regenerative medicine. We previously characterized in vitro human umbilical di-chimeric cells (HUDCs) created via the ex vivo fusion of human umbilical cord blood (UCB) cells derived from two unrelated donors. In this in vivo study, we assessed HUDC safety and biodistribution in the NOD SCID mouse model at 90 days following the systemic intraosseous administration of HUDCs. Twelve NOD SCID mice (*n* = 6/group) received intraosseous injection of donor UCB cells (3.0 × 10^6^) in Group 1, or HUDCs (3.0 × 10^6^) in Group 2, without immunosuppression. Flow cytometry assessed hematopoietic cell surface markers in peripheral blood and the presence of HLA-ABC class I antigens in lymphoid and non-lymphoid organs. HUDC safety was assessed by weekly evaluations, magnetic resonance imaging (MRI), and at autopsy for tumorigenicity. At 90 days after intraosseous cell administration, the comparable expression of HLA-ABC class I antigens in selected organs was found in UCB control and HUDC therapy groups. MRI and autopsy confirmed safety by no signs of tumor growth. This study confirmed HUDC biodistribution to selected lymphoid organs following intraosseous administration, without immunosuppression. These data introduce HUDCs as a novel promising approach for immunomodulation in transplantation.

## 1. Introduction

The field of transplantation represents one of the most remarkable achievements of modern medicine, offering a lifeline to patients facing end-stage organ failure, cancer, or tissue defects. Despite decades of progress driven by advancements in surgical techniques, multidrug immunosuppression therapies, and organ preservation methodologies, a persistent gap persists between organ demand and availability, presenting a global health challenge [1,2]. This scarcity not only restricts access to life-saving treatments but also contributes to significant morbidity and mortality among patients on transplant waiting lists [3]. Moreover, the inherent complexities of solid organ transplantation and the new field of vascularized composite allotransplantation (VCA) poses unique obstacles, encompassing the risk of rejection, graft dysfunction, and the need for lifelong immunosuppression. These challenges underscore the urgent need for innovative strategies within the realm of regenerative medicine, which aims not only to improve transplant outcomes but also to stimulate tissue regeneration and alleviate the burden of organ shortage [4,5].

Currently, the success of VCA and transplantation depends on lifelong multidrug immunosuppression therapy, crucial for preventing graft rejection and ensuring favorable outcomes. However, the burden of adverse effects associated with immunosuppression therapy, including increased risks of metabolic disorders, susceptibility to opportunistic infections, and potential for malignancy growth, underscores the urgent need for alternative therapeutic strategies [6,7,8,9]. In response to these challenges, cell-based therapies have emerged as a promising avenue. By leveraging the immunomodulatory properties of human stem cells and triggering mixed chimerism induction, these novel approaches aim to induce immunomodulation and alleviate the reliance on conventional immunosuppressive therapies [10,11]. 

Recent reports provide new insights on the role of asymmetric T-cell division as the potential implications for immunotherapy [12].

Nevertheless, the translation of these cell-based therapies from preclinical models to clinical practice requires assessment of the maintenance of both donor-specific phenotypes and safety profile. These confirmations are crucial for expanding the clinical applicability of cellular therapies and propelling advancements in the field of regenerative medicine [13,14].

Our laboratory has over 20 years of research experience in chimerism induction and the application of cell-based therapies across various animal models [15,16]. We have successfully developed donor-recipient chimeric cells (DRCCs) via the ex vivo polyethylene glycol (PEG)-mediated cell fusion of bone marrow cells derived from unrelated ACI and Lewis donor rats. Subsequently, we assessed the immunomodulatory properties of DRCC therapy in a fully major histocompatibility complex (MHC)-mismatched model [16], confirming the maintenance of donor-specific hematopoietic phenotypes, successful engraftment of chimeric cells, and increased allograft survival rates without the need for immunosuppression therapy. These promising outcomes provide strong evidence for the potential of chimeric cell-based therapies in overcoming the limitations associated with lifelong immunosuppression therapy.

Based on these encouraging findings in rodent models, we translated cell fusion technology to human hematopoietic progenitor stem cells, including both bone marrow and umbilical cord blood (UCB) cells. Recently, we reported the creation of two novel hematopoietic cell lines: human hematopoietic CD34^+^ chimeric cells (HHCCs) [17] and human umbilical di-chimeric cell (HUDCs) [18]. Through in vitro characterization, we successfully confirmed the viability, genotype, phenotype, safety, and clonogenic properties of both HHCC and HUDC lines, thereby establishing their reliability for future therapeutic approaches in regenerative medicine applications.

To further evaluate the potential clinical applicability of HUDC therapy, this study aimed to assess in vivo the safety and biodistribution of HUDCs tested in the well-established non-obese diabetic/severe combined immunodeficiency (NOD SCID) mouse model following systemic intraosseous administration.

The in vivo validation of HUDC therapy safety in the preclinical NOD SCID mouse model highlighted the immunomodulatory potential of the HUDC line. This innovative approach not only demonstrated the safety of HUDC but also underscored its potential applicability in various fields of regenerative medicine. By providing a cell-based tolerogenic and immunomodulatory strategy, HUDC therapy presents a promising avenue for addressing the challenges inherent to transplantation and advancing the realm of regenerative medicine.

## 2. Materials and Methods

### 2.1. Preparation of UCB Cells and HUDC

**Figure 1:**
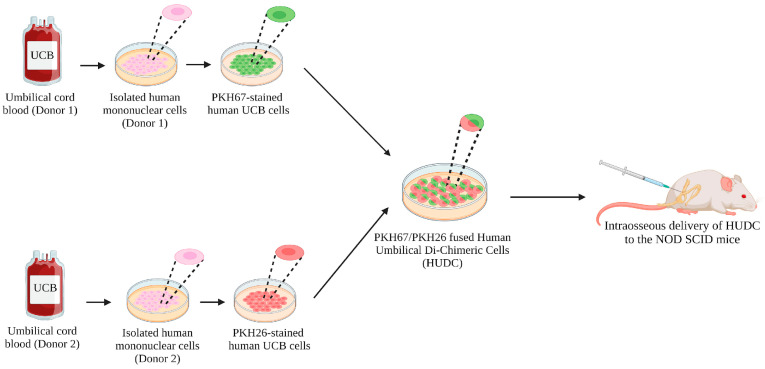
Experimental study design for assessing a novel hematopoietic human umbilical di-chimeric cell (HUDC) line in the non-obese diabetic/severe combined immunodeficiency (NOD SCID) mouse model. Schematic illustration outlining the study design, including the isolation of umbilical cord blood (UCB) cells from two unrelated donors, each labeled with fluorescent markers, PKH67 (green) and PKH26 (red), respectively. The UCB cells were subjected to an ex vivo polyethylene glycol (PEG)-mediated fusion procedure, resulting in the successful creation of HUDC, confirmed by double labeling with PKH67 and PKH26. The subsequent in vivo evaluation aimed to assess the safety and engraftment of HUDC therapy following intraosseous delivery in the NOD SCID mouse model.

Human UCB units were purchased from the Cleveland Cord Blood Bank. The UIC Office for the Protection of Research Subjects has determined that this activity does not meet the definition of research on human subject as defined by the 45 Code of Federal Regulations (CFR) 46.102(f). Due to the nature of the study, no ethical approval or informed consent were required. As previously reported [18], the HUDC cell line was created from human UCB cells derived from two unrelated donors (Figure 1). Briefly, density gradient centrifugation (Lymphoprep™, StemCell™ Technologies, Vancouver, BC, Canada) and anti-human CD235a (Glycophorin A) MicroBeads in conjunction with magnetic-activated cell sorting (MACS^®^, Miltenyi Biotec, Bergisch Gladbach, Germany) were used to isolate and purify UCB-derived cells. Next, the isolated UCB donor cells were either employed directly in vivo as controls or for creating HUDC therapy. The human UCB cells, derived from two unrelated donors, were individually labeled using fluorescence dyes: PKH26 (red) and PKH6 7(green) (MiliporeSigma, Burlington, MA, USA). These labeled cells were mixed in a 1:1 ratio and fused using polyethylene glycol (PEG) 4000 solution (EMD, Burlington, MA, USA), as previously reported [17,18,19,20]. The fluorescence-activated cell sorting (FACS) was conducted employing a BD FACSAria^TM^ II cell sorter (BD Biosciences, Franklin Lakes, NJ, USA) to select the HUDC population based on double PKH26/PKH67 labeling. A total of sixteen fusions were performed, and the average fusion efficacy was at 67.4% ± 3.4% [18].

### 2.2. Mice and Animal Care

This animal study protocol was approved by the Animal Care Committee (ACC number: 16-176, date of approval: 12 January 2016) of the University of Illinois at Chicago, which is accredited by the American Association for the Accreditation of Laboratory Animal Care (AAALAC). All animals received humane care in compliance with the ‘Principles of Laboratory Animal Care’ formulated by the National Society for Medical Research and the ‘Guide for the Care and Use of Laboratory Animal Resources’ published by the US National Institutes of Health. In this experimental study, a total of twelve male NOD SCID mice (strain NOD.Cg-Prkdc^scid^/J, RRID: IMSR_JAX:001303), purchased from the Jackson Laboratory (Bar Harbor, ME, USA), were used. The animals were housed in an accredited animal facility at the University of Illinois at Chicago with access to rodent food and water ad libitum and maintained on a twelve-hour light/dark cycle.

### 2.3. Study Design and Experimental Groups

Prior to study initiation, twelve NOD SCID mice were selected, ear-tagged, randomized, and divided into two experimental groups (*n* = 6/group) based on the type of cells administered. Based on our prior experience [21], both groups received equivalent doses of cellular therapies via intraosseous delivery. Group 1, serving as the control, received 3.0 × 10^6^ of mixed UCB cells derived from two unrelated human donors (1.5 × 10^6^ cells per donor) and Group 2 received a comparable dose of 3.0 × 10^6^ of fused HUDC. None of the mice received immunosuppression therapy throughout the study. After the injection of mixed UCB donor cells or HUDC, all treated animals were observed daily for any local signs of infection, edema, or hematoma.

### 2.4. Systemic Intraosseous Transplantation of UCB Cells and HUDC

The intraosseous transplantation of the mixed UCB control cells or HUDC was performed directly into the right femoral bone of the NOD SCID mice, as previously reported [15,20]. Briefly, mice were anesthetized with the inhalation of Isoflurane 1.5–2.5% and a subcutaneous injection of 1 mL/kg Buprenorphine. Following a 1 cm incision over right mid-femoral level, the skin and overlying muscles were dissected to expose the femoral bone. To reduce the risk of hyperpressure during injection and to allow a better distribution of the cell suspension in the intraosseous compartment, 60 μL of recipient bone marrow was aspirated using a 0.5 mL syringe and a 30-gauge needle (320468, BD Ultra-Fine™ Insulin Syringes, Becton Dickinson, Franklin Lakes, NJ, USA) prior to injection. Next, the injection of UCB donor cells or HUDCs, suspended in 60 μL of normal saline solution, was gently performed to avoid hyperpressure with a 0.5 mL syringe using a 30-gauge needle (320468, BD Ultra-Fine™ Insulin Syringes, Becton Dickinson) through a hole created in the femoral bone with a 30-gauge drill, which was then sealed with bone wax (Medeline Industries, Mundelein, IL, USA) to prevent any cell leakage. Next, the muscles and skin were reapproximated and the wound was closed with 4-0 nylon monofilament non-absorbable suture (Ethicon Inc., Raritan, NJ, USA). Post-operatively, the mice recovered in a heated environment and received necessary care before returning to the colony.

### 2.5. Blood Sampling Procedure

Samples of peripheral blood were collected at 90 days following systemic intraosseous administration of mixed UCB donor cells or HUDC. During the blood sampling procedure, mice were anesthetized with 1.5–2.5% Isoflurane inhalation. Blood samples were collected in ethylenediaminetetraacetate collection tubes (BD Microtainer^®^, Becton Dickinson) and immediately processed by the accredited Biologic Resources Laboratory at the University of Illinois at Chicago, for complete blood counts, including white blood cells (WBC), lymphocytes, neutrophils, monocytes, eosinophils, and basophils.

### 2.6. Flow Cytometry Assessment of Hematopoietic Cell Surface Markers Expression 

At 90 days following systemic intrasseous administration of mixed UCB donor cells or HUDC, the collected blood samples were washed and suspended in a phosphate-buffered saline (PBS) staining buffer containing 1% bovine serum albumin (BSA). To block nonspecific binding, the blood samples were treated with mouse BD Fc Block^TM^ Reagent (BD Biosciences) for 5 min, subsequently treated with anti-human monoclonal antibodies (mAbs) at saturating concentrations and incubated for 30 min on ice. The following mAbs were used: CD4 (BD Pharmingen^TM^ APC-Cy^TM^7, RRID:AB_398521, BD Biosciences), CD19 (APC/Cyanine7, RRID:AB_314248, BioLegend, San Diego, CA, USA), CD20 (BD Pharmingen^TM^ APC Mouse Anti-Human CD20, RRID:AB_398670, BD Biosciences), CD45 (Brilliant Violet 570^TM^, RRID:AB_10899568, BioLegend), and HLA-ABC class I (BD Pharmingen^TM^ APC, RRID:AB_398603, BD Biosciences) to measure the respective marker expression level. Following mAbs incubation, all samples were washed twice, resuspended in a staining buffer, and analyzed by FC (Gallios, Beckman Coulter, Brea, CA, USA) employing a BD LSRFortessa^TM^ Cell Analyzer (RRID:SCR_018655, BD Biosciences) and Flowjo^TM^ software (For Mac, Version vX.0.7., RRID:SCR_008520, Becton Dickinson) to assess the in vivo phenotype of peripheral blood samples following HUDC administration.

### 2.7. Assessment of HLA-ABC Class I Antigen Expression in the Organs

To assess the biodistribution and presence of human cell-based therapy within the selected organs of NOD SCID mice, FC analysis was conducted in the selected lymphoid organs (bone marrow, lymph nodes, spleen, and thymus), and non-lymphoid organs (brain, intestine, kidney, liver, lung, and peripheral blood) at 90 days following systemic intraosseous delivery. To fulfill this aim, we selected the anti-human mAb HLA-ABC class I (BD Pharmingen^TM^ APC, RRID:AB_398603, BD Biosciences), which specifically targets the HLA-ABC class I antigen on human cells, to assess the human origin of the cells within the selected organs. The cell solution isolated from the respective organs was stained in PBS containing 1% BSA, with anti-human mAb, and incubated for 40 min at 4 °C in the dark. The samples were washed with PBS supplemented with 1% BSA, fixed with 4% of paraformaldehyde, washed with PBS, and then assessed by a flow cytometer (Gallios, Backman Coulter, CA, USA) using the Flowjo^TM^ (For Mac, Version vX.0.7., RRID:SCR_008520, Becton Dickinson) software.

### 2.8. Clinical Assessment and Evaluation at Autopsy

To assess local safety and tolerance following the systemic intraosseous administration of mixed UCB control cells or HUDC, mice were observed on daily basis during the initial two weeks for the presence of any side effects at the injection site, including parameters such as bruising, redness, inflammation, infection, hematoma, and wound dehiscence, as well as acute or delayed signs of immune responses, such as anaphylactic reaction, edema, erythema, and inflammatory response. Daily clinical observations and examination of NOD SCID mice for any changes in activity, posture, or fur coverage were performed by an experienced board-certified veterinarian from the Veterinary Diagnostic Laboratory at the University of Illinois at Chicago. Moreover, animals underwent palpation examinations three times per week to detect any potential growth formations or abnormalities. At the 90-day study endpoint, an autopsy and full organ necropsy were performed by an experienced board-certified veterinarian to examine organ anatomy and size, and to assess any evidence of pathology, tumors, or the presence of fluids within the evaluated organs and body cavities.

### 2.9. Magnetic Resonance Imaging (MRI) Scanning

To further confirm the lack of tumor growth and identify any organ abnormalities in the NOD SCID mice, MRI scanning was conducted employing a preclinical scanner (Agilent/Varian 9.4 T, Agilent Technologies, Santa Clara, CA, USA) with a 39 mm proton volume coil. In preparation for MRI scanning, mice were initially induced with Isoflurane 2% and then maintained under Isoflurane 1–1.5% in 100% oxygen throughout the procedure. Body temperature was continuously monitored by employing a rectal probe and maintained at 37.5 °C by regulating warm air flow into the scanner bore. Temperature and respiration rate were monitored using an MRI-compatible physiological monitoring system (Model 1025, SA Instruments Inc., Stony Brook, NY, USA) during the scanning procedure. Each mouse underwent scanning in three sections: brain, chest, and abdomen at 90 days following the mixed UCB donor cells or HUDC administration. The T1-weighted images were acquired for tumor detection in solid organs, which was made possible by greater soft tissue resolution. The scan parameters for T1-weighted images included the following parameters: a field of view of 40 × 40 mm^2^, a matrix size 128 × 128, a repetition time of 550 ms, and an echo time of 28 ms.

### 2.10. Statistical Analysis

Statistical analysis was performed using GraphPad Prism 9.5.0 (RRID:SCR_002798, Dotmatics, Boston, MA, USA) software. Assessments were performed in independent experiments with mixed UCB cells from two human donors as reference controls. Data are presented as mean ± standard error of the mean (SEM). Statistical differences between respective groups were assessed using two-way analysis of variance (ANOVA); *p* values below 0.05 were considered significant.

## 3. Results

### 3.1. Confirmation of Comparable Distribution of WBC Cell Subpopulations at 90 Days after the Systemic Intraosseous Administration of UCB Cells and HUDC

**Figure 2:**
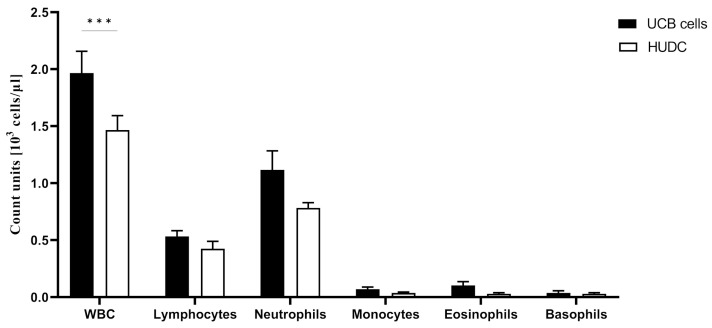
Impact of intraosseous administration of human cellular therapies on white blood cell (WBC) counts in the peripheral blood of NOD SCID mice at the 90-day study endpoint. The UCB cell group exhibited the highest WBC values compared to the HUDC group. However, comparable values for WBC subpopulations, including lymphocytes, neutrophils, monocytes, eosinophils, and basophils, were observed, indicating a similar cell distribution pattern following the intraosseous administration of UCB cells and HUDC. Data are presented as mean ± SEM. A two-way analysis of variance (ANOVA) test for group comparison was used to define statistical significance, *** *p* < 0.001.

Peripheral blood cells population counts, including WBC, lymphocytes, neutrophils, monocytes, eosinophils, and basophils values at the 90-day study endpoint following intraosseous administration of UCB cells or HUDC, are presented in Figure 2. At the 90-day study endpoint, the WBC count exhibited a significant elevation following UCB cells administration when compared to HUDC (UCB cells: 1.97% ± 0.19%, vs. HUDC: 1.47% ± 0.13%, *p* < 0.001, *n* = 6/group). However, the comparative analysis of WBC subpopulation counts revealed no significant differences in cell levels for lymphocytes (UCB cells: 0.53% ± 0.05%, vs. HUDC: 0.43% ± 0.06%), neutrophils (UCB cells: 1.12% ± 0.17%, vs. HUDC: 0.78% ± 0.05%), monocytes (UCB cells: 0.07% ± 0.02%, vs. HUDC: 0.04% ± 0.01%), eosinophils (UCB cells: 0.10% ± 0.03%, vs. HUDC: 0.03% ± 0.01%), and basophils (UCB cells: 0.04% ± 0.02%, vs. HUDC: 0.03% ± 0.01%) at 90 days following intraosseous administration. These findings confirmed the comparable immune cell distribution pattern for both granulocytes and agranulocytes at the 90-day study endpoint following UCB cells and HUDC administration.

### 3.2. Confirmation of the HUDC Hematopoietic Phenotype Maintenance at 90 Days after Systemic Intraosseous Administration

**Figure 3:**
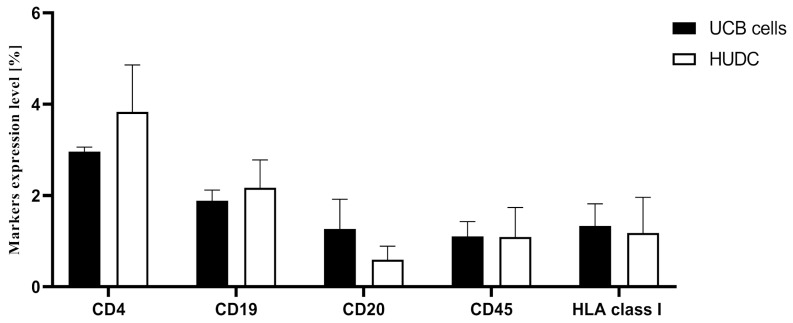
Assessment of hematopoietic cell surface markers in peripheral blood at day 90 following intraosseous administration of UCB cells and HUDC to the NOD SCID mice. The comparative analysis of hematopoietic cell surface markers (CD4, CD19, CD20, CD45, and HLA class I) revealed comparable levels of hematopoietic cell surface markers expression following intraosseous administration of UCB cells delivery and HUDC, confirming the maintenance of UCB parent cells phenotype in the fused HUDC. Data are presented as mean ± SEM. A two-way ANOVA test for group comparison was used to define statistical significance.

The FC analysis of CD4, CD19, CD20, CD45, and HLA class I surface marker levels confirmed the comparable percentage values of human-derived hematopoietic and stem cell surface markers following both UCB cells and HUDC administration (Figure 3). There were no significant differences in the percentages of markers expression level of the following: CD4 (UCB cells: 2.97% ± 0.10%, vs. HUDC: 3.84% ± 1.03%), CD19 (UCB cells: 1.89% ± 0.23%, vs. HUDC: 2.18% ± 0.61%), CD20 (UCB cells: 1.27% ± 0.65%, vs. HUDC: 0.60% ± 0.30%), CD45 (UCB cells: 1.11% ± 0.33%, vs. HUDC: 1.10% ± 0.65%), and HLA class I (UCB cells: 1.34% ± 0.49%, vs. HUDC: 1.18% ± 0.78%). The comparable expression of human-derived hematopoietic cell surface markers in the peripheral blood of the NOD SCID mice after systemic intraosseous HUDC delivery, when compared to the marker expression after UCB control cell administration, confirmed hematopoietic phenotype maintenance following both cell-based therapies.

### 3.3. Confirmation of HUDC Biodistribution to the Selected Organs by Presence of the HLA-ABC Class I Antigen at 90 Days after Systemic Intraosseous Administration

**Figure 4:**
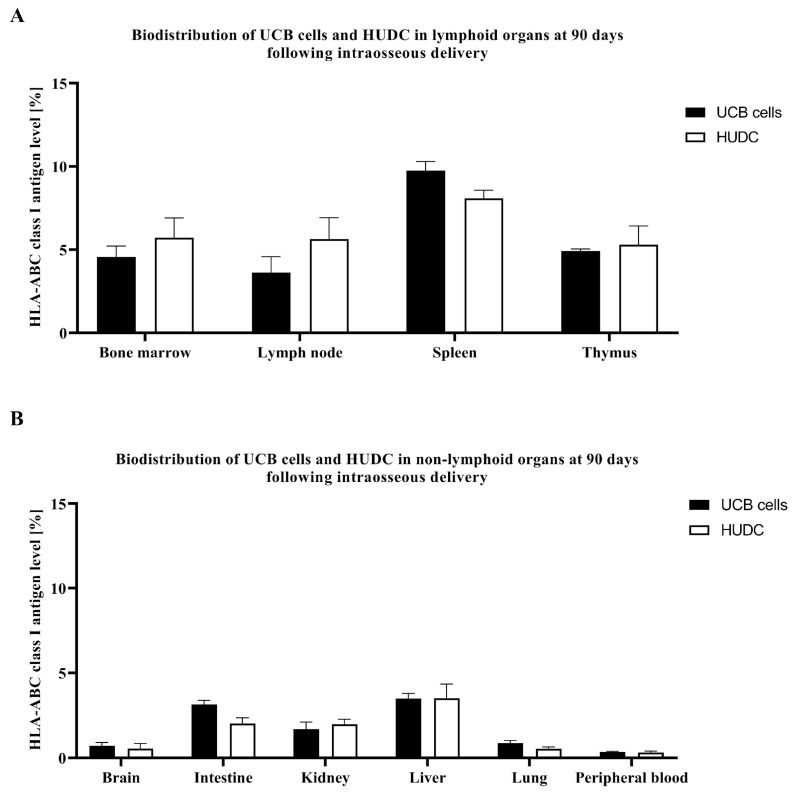
Evaluation of HLA-ABC class I antigen expression in both (**A**) lymphoid and (**B**) non-lymphoid organs at 90 days following the systemic intraosseous administration of UCB cells and HUDC to the NOD SCID mice. (**A**) In the lymphoid organs, the expression of HLA-ABC class I antigen was comparable following administration of both UCB cells and fused HUDCs. Notably, both groups revealed higher HLA-ABC class I antigen expressions in the spleen compared to other lymphoid organs. (**B**) The minimal expression of HLA-ABC class I antigen was observed in non-lymphoid organs following HUDC administration when compared to the UCB-cell-injected controls. Particularly, the lowest values of HLA-ABC class I antigen expression were observed in peripheral blood, brain, and lung tissues. Data are presented as mean ± SEM. A two-way ANOVA test for group comparisons was used to define statistical significance.

At the study endpoint of 90 days after intraosseous administration, the safety of HUDC therapy was assessed by detection of HUDC biodistribution when compared to UCB control cells. The assessment focused on the expression of human HLA-ABC markers in selected organs, including lymphoid (bone marrow, lymph nodes, spleen, and thymus) and non-lymphoid (brain, intestine, kidney, liver, lung, and peripheral blood) organs. The FC comparative analysis revealed comparable levels of HLA-ABC class I antigens expressions after HUDC administration when compared to UCB cells in the lymphoid organs, as shown in bone marrow (HUDC: 5.72% ± 1.18%, vs. UCB cells: 4.57% ± 0.65%), thymus (HUDC: 5.30% ± 1.13%, vs. UCB cells: 4.92% ± 0.12%), lymph nodes (HUDC: 2.60% ± 0.11%, vs. UCB cells: 3.63% ± 0.95%), and spleen (HUDC: 8.09% ± 0.48%, vs. UCB cells: 9.75% ± 0.54%) (Figure 4A), as well as in the non-lymphoid organs, as shown in peripheral blood (HUDC: 0.32% ± 0.08%, vs. UCB cells: 0.35% ± 0.04%), brain (HUDC: 0.55% ± 0.30%, vs. UCB cells: 0.72% ± 0.20%), kidney (HUDC: 1.98% ± 0.29%, vs. UCB cells: 1.70% ± 0.41%), liver (HUDC: 3.51% ± 0.84%, vs. UCB cells: 3.49% ± 0.30%), lung (HUDC: 0.54% ± 0.10%, vs. UCB cells: 0.86% ± 0.16%), and intestine (HUDC: 2.02% ± 0.35%, vs. UCB cells: 3.15% ± 0.24%) (Figure 4B). The comparable expression of HLA-ABC class I antigens, specific for human cells, in the selected lymphoid and non-lymphoid organs demonstrated the comparable migration pattern of HUDC when compared to UCB cells of lymphoid and non-lymphoid organs in NOD SCID mice.

### 3.4. Confirmation of Long-Term HUDC Safety at Autopsy Assessed at 90 Days after Systemic Intraosseous Administration

Throughout the entire study, all animals presented normal activity levels, consistent weight gain, displayed normal fur condition, and showed no signs of tumor-like growths, including at the injection site. No signs of bruising, redness, inflammation, infection, or wound dehiscence were detected over the entire 90-day observation period. At the 90-day study endpoint, autopsy evaluation showed no concerning changes, such as tumor formation, organ enlargement, organ shrinkage, inflammation signs, or the presence of fluid within body cavities. The macroscopic evaluation of harvested organs revealed consistent size, appearance, or texture in animals treated with HUDC therapy compared to the UCB cell control group following systemic intraosseous delivery.

### 3.5. Confirmation of Long-Term HUDC Safety by Lack of Tumor Formation on MRI Scans at 90 Days after Systemic Intraosseous Administration

**Figure 5:**
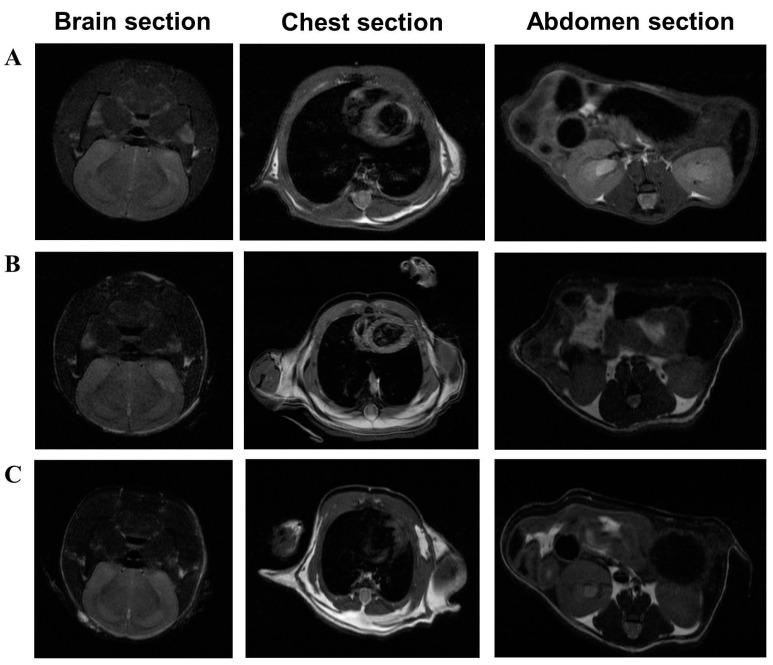
Confirmation of HUDC safety due to the lack of tumor formation at 90 days after systemic intraosseous administration assessed by magnetic resonance imaging (MRI) in the NOD SCID mice. The safety assessment of HUDC therapy involved a comparative analysis of T1-weighted brain, chest, and abdomen scans at 90 days after the systemic intraosseous administration of (**A**) negative controls (naïve NOD SCID mouse), (**B**) UCB cells, and (**C**) HUDC. No tumorous-like structures were observed up to 90 days after cell delivery. T1-weighted scan parameters included a field of view of 40 × 40 mm^2^, matrix size of 128 × 128, repetition time of 550 ms, and echo time of 28 ms.

At the 90-day study endpoint, MRI scans of the brain, chest, and abdomen were assessed by a board-certified radiologist. Comparative analysis with non-injected NOD SCID control mice (Figure 5A) and those injected with UCB cells (Figure 5B) and HUDC (Figure 5C) revealed no growths or abnormalities on T1-weighted images. These results confirm the lack of tumorigenicity during the 90-day observation period, thereby affirming the long-term safety of HUDC therapy. Thus, these findings confirm the non-tumorigenic nature of HUDC therapy administered intraosseous to NOD SCID mice during long-term follow-up.

## 4. Discussion

The field of transplantation and regenerative medicine faces a significant global challenge characterized by a limited number of safe and effective therapies across various regenerative fields. Despite notable advancements in surgical techniques, tissue engineering, and immunosuppression protocols, the persistent shortage of donor organs and viable treatment options remains a critical challenge [22,23,24]. This scarcity not only prolongs waiting periods for patients but also exacerbates their medical conditions, leading to uncertainty and frequently unfavorable prognoses and outcomes [25,26]. Moreover, the intricate interplay of immunological factors presents obstacles, increasing the risk of graft rejection and requiring long-term immunosuppressive regimens that generate well-known adverse effects [27,28]. While advancements in organ preservation techniques and the establishment of national Organ Procurement and Transplantation networks have improved access to transplantable organs to some extent, the persistent demand continues to exceed the available supply, supporting the urgent need for innovative therapeutic approaches [29,30,31]. 

Within this overarching challenge, solid organ and VCA transplantation are emerging as particularly critical concerns. Solid organ transplantation faces distinct challenges related to organ compatibility, ischemia–reperfusion injury, and the imperative for lifelong immunosuppression [32,33,34]. Similarly, VCA transplantation introduces additional complexities due to the unique requisites of VCA organs, such as the face, hands, or uterus, and the inherent risk of rejection [35,36,37]. Addressing these challenges requires efforts to introduce and develop novel therapeutic approaches capable of enhancing graft survival, promoting tissue regeneration, and improving long-term outcomes across the diverse spectrum of transplantation and regenerative medicine.

The scientific literature highlights the significant potential of umbilical cord blood (UCB) as a valuable source of stem cells with tolerogenic and immunomodulatory properties [38,39,40]. UCB serves as a source of mesenchymal stem cells [41,42,43] and progenitor hematopoietic cells [44,45,46]. Notably, UCB presents distinct advantages over bone marrow transplants, including lower immunogenicity levels [47,48,49] and reduced requirements for HLA matching [50,51,52], thereby broadening the pool of potential donors and improving accessibility to transplantation and tissue regeneration.

Furthermore, extensive research has validated the collection, banking, and cryopreservation of UCB for long-term storage, highlighting its potential for therapeutic applications [53,54,55,56,57]. Importantly, cryopreservation has been demonstrated not to significantly alter the clonogenic or tolerogenic properties of UCB cells [58,59,60], further enhancing its utility in clinical settings. Intriguingly, UCB-derived therapies retain their regenerative properties, facilitating the restoration of functional hematopoiesis even in cases of compromised hematopoietic function following radiotherapy, as evidenced in rodent models [61,62].

The recognized potential of UCB in therapeutic applications, coupled with its successful preservation through cryopreservation without compromising its properties, suggests a promising avenue for medical advancements. These challenges present new opportunities for the exploration and development of novel therapeutic strategies applicable to the growing field of reconstructive transplantation and tissue regeneration.

We have already made significant strides in addressing the pressing needs of VCA transplants and regenerative medicine. Over the past two decades, our laboratory has introduced several innovative cell-based therapies aimed at developing donor-specific chimerism and inducing immunomodulation in experimental models without the need for lifelong immunosuppressive therapy.

The rationale behind creating donor-recipient chimeric cells is based on the published transplantation studies, which have demonstrated that the establishment of donor-specific cellular chimerism correlates with enhanced acceptance of solid organ transplants, leading to donor-specific tolerance. Notable research by Starzl et al. has highlighted this phenomenon in liver transplantation [63,64,65], and kidney transplantation [66,67], thereby motivating the creation and systemic administration of donor-recipient chimeric cells. This approach aims to induce chimerism between the organ donor and the recipient, thereby mitigating cell rejection and promoting long-term engraftment without the need for immunosuppression.

Our previous studies laid the groundwork for the development of novel human hematopoietic cell lines through an ex vivo PEG-mediated cell fusion procedure [16,17,18,19]. Utilizing this protocol, we successfully confirmed the creation of the human umbilical di-chimeric cell (HUDC) line by fusing human UCB cells derived from two unrelated human donors. Through in vitro evaluation, we confirmed the di-chimeric state, absence of genotoxicity, preservation of hematopoietic phenotype, and maintenance of clonogenic properties of the HUDC following cell fusion [18].

Given the intricate nature of cell-based therapy within the transplantation field, a variety of animal models have been investigated to gain valuable insights into the immunomodulation, induction of graft tolerance, and factors influencing tissue regeneration. Among these models, rodent models have emerged as the most popular choice for preclinical studies due to the relatively low housing cost, ease of maintenance, and extensive genetic and physiological similarities to humans [14,68,69].

We have previously reported the proof-of-concept studies testing a new chimeric cell line of the dystrophin expressing chimeric (DEC) cells, created by fusion of human myoblasts derived from duchenne muscular dystrophy (DMD) patients and normal healthy donors. This novel stem-cell-based therapy was tested in the immunocompromised *mdx/scid* mouse model of DMD [70]. The in vitro assessments confirmed that human DEC cells displayed the phenotype and genotype of donor’s parent cells, expressed dystrophin, and maintained proliferation and myogenic differentiation potential. In vivo studies confirmed the long-term engraftment of human DEC cells, which correlated with restoration of dystrophin expression and significant improvement of muscle function after intraosseous transplantation to the *mdx/scid* mice. Therefore, we tested and confirmed the concept of human chimeric cells of myoblast origin to be safe and efficacious in the immunocompromised murine model of DMD. Based on these encouraging outcomes on safety and efficacy tested in the preclinical *mdx/scid* mouse model of DMD, we have tested DEC cell therapy in the first-in-human study in DMD patients. Specifically, we have confirmed both the safety and efficacy of DEC chimeric myoblasts in DMD patients up to 12 months [71,72] and currently up to 24 months after systemic intraosseous administration to DMD patients without the need for immunosuppression. These promising outcomes underscore the feasibility of scaling up chimeric cell propagation under the good manufacturing practice (GMP) conditions, as confirmed in our pilot clinical study [71,72].

Encouraged by the promising findings from the in vitro assessment of HUDC therapy and the clinical study of chimeric DEC cell therapy tested in DMD patients, this study conducted a series of in vivo experiments utilizing the NOD SCID mouse model, a well-established model to study the biodistribution and safety of human stem cells. These experiments were designed to evaluate critical aspects, including the viability of fused cells, safety profiles, and biodistribution, all essential in assessing the therapeutic efficacy of HUDC therapy. To prove the concept of engraftment and safety of cells of human origin, it was essential to conduct the preclinical studies in an immunocompromised animal model to check the biodistribution and safety of human cells as reported by other investigators [73,74,75,76]. Therefore, to ensure that human cells will not be rejected by the recipient’s immune system, this preclinical study was tested in the immunocompromised NOD SCID mouse model. The NOD SCID mouse model provides a unique approach to investigate new cell-based therapies, employing a specific manufacturing protocol aligning with the regulatory prerequisites. Other researchers utilize the NOD SCID mouse model to investigate the engraftment of human-origin stem cells [77,78,79,80,81,82,83]. The regulatory prerequisites mandates that preclinical efficacy studies maintain the same type of cellular therapy intended for the future clinical trials. Moreover, this animal model is recognized as a preclinical proof-of-concept model by regulatory bodies and is considered as a standard experimental model in transplantation research [83,84,85,86,87]. Thus, using the NOD SCID mouse model in our study adheres to these regulatory prerequisites, aiming to facilitate the transition from the preclinical studies to the clinical trials.

First, we confirmed a comparable engraftment of different WBC subpopulations between the UCB control cells and HUDC at 90 days after systemic intraosseous delivery. These findings supported the engraftment of HUDC, as evidenced by consistent distribution of blood cells.

Next, we confirmed the presence of hematopoietic cell surface markers, including CD4, CD19, CD20, CD45, and HLA class I, in the peripheral blood following administration of both the UCB parent cells and HUDC. The expression of the hematopoietic cell surface markers recorded following the administration of fused HUDC cells was comparable with the hematopoietic markers assessed after transplantation of the non-fused parent UCB cells. These findings confirmed the donor–recipient hematopoietic phenotype and chimeric state of HUDC, further suggesting that neither the fusion procedure nor the in vivo conditions adversely affected the expression pattern of the hematopoietic cell surface markers on HUDC after cell fusion. Furthermore, the stability and maintenance of the hematopoietic phenotype of HUDC in peripheral blood following systemic intraosseous delivery confirms the safety of HUDC therapy and its potential immunomodulatory properties.

Analysis of HLA-ABC class I antigen expression 90 days after the administration of both UCB cells and HUDC revealed lower expression in the non-lymphoid organs, including the brain, intestine, kidney, liver, lung, and peripheral blood, when compared to the higher expression observed in lymphoid organs, including the bone marrow, lymph node, spleen, and thymus. These findings provide valuable insights into the preferential biodistribution of HUDC therapy to the lymphoid organs, comparable to the biodistribution pattern observed for the UCB parent cells, following systemic intraosseous administration. 

Furthermore, the analysis of lungs revealed the negligible expression of human HLA-ABC class I antigens after transplantation of both UCB cells and HUDC. This observation strengthens the long-term safety profile of the systemic intraosseous administration of both cell lines. Additionally, this provides additional rationale for selecting intraosseous route of administration for UCB-cell-based therapies over intravenous administration, which has been repeatedly associated with the pulmonary first-pass effect, leading to cell entrapment in the lungs, as reported following administration of mesenchymal-stem-cell-based therapies [88,89,90,91]. Moreover, beyond the differences in the size of stem cells of bone marrow, mesenchymal, or UCB origin, the scientific literature highlights the disparity in the profiles of cell-surface-adhesion molecules, leading to distinct lung adherence rates. Notably, UCB-derived cells exhibit faster lung clearance rates compared to the bone marrow-derived cells [92,93].

The assessment of HUDC therapy safety by weekly clinical observations, revealed no impact on the general animal conditions as evidenced by normal activity levels, posture, food consumption, and the lack of weight fluctuations throughout the entire observation period. Furthermore, at the 90-day study endpoint, the macroscopic evaluation of the harvested organs during autopsy revealed no evidence of pathology, tumors, or tumor-like structures. The local and systemic safety of the intraosseous administration of HUDC was confirmed by the absence of local or generalized side effects over 90 days of follow-up.

Moreover, the T1-weighted MRI scanning of the brain, chest, and abdomen confirmed the lack of tumor formation throughout the entire follow-up period of 90 days. This is in contrast to reports on tumor formation following the administration of other stem-cell-based therapies, including embryonic stem cells (ESCs) and induced pluripotent stem cells (iPSCs), which limits the clinical applications of these cells [94,95,96,97,98]. Overall, our findings confirm the long-term safety and the lack of tumorigenicity of HUDC therapy after intraosseous administration.

There are some limitations of this study that should be addressed. We have tested HUDC therapy in the NOD SCID mouse model since it provides distinct advantages for assessing novel cell-based therapies of human origin. Notably, this immunocompromised murine model facilitated a comprehensive evaluation of human cell safety and biodistribution to the lymphoid and non-lymphoid organs within a living system. In addition, the use of the NOD SCID mouse model offers a unique approach to evaluating human hematopoietic chimeric cell engraftment in peripheral blood [99,100], employing the same manufacturing protocol intended for future clinical applications. While the NOD SCID mouse model serves as a valuable tool, it may not fully replicate the complexity of immune reactions.

Nevertheless, despite these limitations, this study lays the groundwork for further investigation of chimeric cell therapies, particularly their immunomodulatory role in tolerance induction across various transplantation fields, including bone marrow, solid organs, and VCA. These findings hold the potential for developing novel therapeutic approaches that could alleviate the requirement for lifelong immunosuppression protocol.

To the best of our knowledge, this study represents the first report on the biodistribution and safety profiles of the novel UCB-based hematopoietic HUDC lines, assessed in the NOD SCID mouse model. In light of these outcomes, HUDC therapy demonstrates promising potential to modulate the immune system with reduced or limited reliance on lifelong immunosuppressive therapy in the growing field of reconstructive transplantation and tissue regeneration. Moreover, this pioneering therapeutic approach addresses the pressing challenge of donor shortage, ultimately providing unlimited access to HUDC therapy.

## 5. Conclusions

This study successfully confirmed the safety of the systemic intraosseous administration of HUDC therapy without the need for immunosuppression, as evidenced by clinical observations, autopsy, and MRI scanning. Additionally, the comparable biodistribution of both the UCB parent cells and the created HUDC cells to the selected lymphoid and non-lymphoid organs following systemic intraosseous administration further confirmed the safety profile of both cell-based therapies. Therefore, HUDC therapy emerged as a promising cell-based tolerogenic and immunomodulatory strategy, applicable for a variety of transplantation fields without the need for lifelong immunosuppression.

## Data Availability

The data presented in this study are included in this published article, further inquiries can be directed to the corresponding author.

## References

[1] Meirelles Júnior R.F., Salvalaggio P., Rezende M.B., Evangelista A.S., Guardia B.D., Matielo C.E., Neves D.B., Pandullo F.L., Felga G.E., Alves J.A. (2015). Liver transplantation: History, outcomes, and perspectives. Einstein.

[2] Tonsho M., Michel S., Ahmed Z., Alessandrini A., Madsen J.C. (2014). Heart transplantation: Challenges facing the field. Cold Spring Harb. Perspect. Med..

[3] Hsiao S., Khush K.K. (2022). Donor selection for multiorgan transplantation. Curr. Opin. Organ Transplant..

[4] Cai S., Chandraker A. (2019). Cell Therapy in Solid Organ Transplantation. Curr. Gene Ther..

[5] Edgar L., Pu T., Porter B., Aziz J.M., La Pointe C., Asthana A., Orlando G. (2020). Regenerative medicine, organ bioengineering and transplantation. Br. J. Surg..

[6] Gordon C.R., Avery R.K., Abouhassan W., Siemionow M. (2011). Cytomegalovirus and other infectious issues related to face transplantation: Specific considerations, lessons learned, and future recommendations. Plast. Reconstr. Surg..

[7] Petruzzo P., Dubernard J.M. (2011). The International Registry on Hand and Composite Tissue allotransplantation. Clin. Transpl..

[8] Messner F., Etra J.W., Dodd-O J.M., Brandacher G. (2019). Chimerism, Transplant Tolerance, and Beyond. Transplantation.

[9] Yang J.H., Johnson A.C., Colakoglu S., Huang C.A., Mathes D.W. (2021). Clinical and preclinical tolerance protocols for vascularized composite allograft transplantation. Arch. Plast. Surg..

[10] Zhang W., Wang Y., Zhong F., Wang X., Sucher R., Lin C.H., Brandacher G., Solari M.G., Gorantla V.S., Zheng X.X. (2023). Donor derived hematopoietic stem cell niche transplantation facilitates mixed chimerism mediated donor specific tolerance. Front. Immunol..

[11] Mengrelis K., Muckenhuber M., Wekerle T. (2023). Chimerism-based Tolerance Induction in Clinical Transplantation: Its Foundations and Mechanisms. Transplantation.

[12] Kaminskiy Y., Ganeeva I., Chasov V., Kudriaeva A., Bulatov E. (2024). Asymmetric T-cell division: Insights from cutting-edge experimental techniques and implications for immunotherapy. Front. Immunol..

[13] Mathes D.W., Chang J., Hwang B., Graves S.S., Storer B.E., Butts-Miwongtum T., Sale G.E., Storb R. (2014). Simultaneous transplantation of hematopoietic stem cells and a vascularized composite allograft leads to tolerance. Transplantation.

[14] Huelsboemer L., Kauke-Navarro M., Reuter S., Stoegner V.A., Feldmann J., Hirsch T., Kueckelhaus M., Dermietzel A. (2023). Tolerance Induction in Vascularized Composite Allotransplantation-A Brief Review of Preclinical Models. Transpl. Int..

[15] Siemionow M., Zielinski M., Ozmen S., Izycki D. (2005). Intraosseus transplantation of donor-derived hematopoietic stem and progenitor cells induces donor-specific chimerism and extends composite tissue allograft survival. Transplant. Proc..

[16] Cwykiel J., Madajka-Niemeyer M., Siemionow M. (2021). Development of Donor Recipient Chimeric Cells of bone marrow origin as a novel approach for tolerance induction in transplantation. Stem Cell Investig..

[17] Siemionow M., Brodowska S., Różczka K., Roesler C. (2022). Creation of human hematopoietic chimeric cell (HHCC) line as a novel strategy for tolerance induction in transplantation. Stem Cell Investig..

[18] Siemionow M., Cwykiel J., Chambily L., Gacek S., Brodowska S. (2023). Novel Human Umbilical Di-Chimeric (HUDC) cell therapy for transplantation without life-long immunosuppression. Stem Cell Investig..

[19] Siemionow M., Cwykiel J., Brodowska S., Chambily L. (2023). Human Multi-Chimeric Cell (HMCC) Therapy as a Novel Approach for Tolerance Induction in Transplantation. Stem Cell Rev. Rep..

[20] Siemionow M., Chambily L., Cwykiel J. (2024). Biodistribution and safety of Human Multi-Chimeric Cells (HMCC) After Systemic Intraosseous and Intravenous Administration in the Experimental Mouse Model. Stem Cells Dev..

[21] Cwykiel J., Jundzill A., Klimczak A., Madajka-Niemeyer M., Siemionow M. (2021). Donor Recipient Chimeric Cells Induce Chimerism and Extend Survival of Vascularized Composite Allografts. Arch. Immunol. Ther. Exp..

[22] Abouna G.M. (2008). Organ shortage crisis: Problems and possible solutions. Transplant. Proc..

[23] Lewis A., Koukoura A., Tsianos G.I., Gargavanis A.A., Nielsen A.A., Vassiliadis E. (2021). Organ donation in the US and Europe: The supply vs demand imbalance. Transplant. Rev..

[24] Anderson D.J., Locke J.E. (2023). Progress towards solving the donor organ shortage. Nat. Rev. Nephrol..

[25] Anthony S.J., Annunziato R.A., Fairey E., Kelly V.L., So S., Wray J. (2014). Waiting for transplant: Physical, psychosocial, and nutritional status considerations for pediatric candidates and implications for care. Pediatr. Transplant..

[26] Salerno C., Pack Q.R., Jurkowski B., McAnally K., Dejong C., Ahmad F.S., Lagu T. (2023). “I Just Wanted Nothing More Than to Get in a Real Shower”: Patient Experience of the Inpatient Wait for a Heart Transplant. J. Card. Fail..

[27] Wojciechowski D., Wiseman A. (2021). Long-Term Immunosuppression Management: Opportunities and Uncertainties. Clin. J. Am. Soc. Nephrol..

[28] Rostaing L., Jouve T., Terrec F., Malvezzi P., Noble J. (2023). Adverse Drug Events after Kidney Transplantation. J. Pers. Med..

[29] Nguyen J.H., Bonatti H., Dickson R.C., Hewitt W.R., Grewal H.P., Willingham D.L., Harnois D.M., Schmitt T.M., Machicao V.I., Ghabril M.S. (2009). Long-term outcomes of donation after cardiac death liver allografts from a single center. Clin. Transplant..

[30] Vandermeulen M., Erpicum P., Weekers L., Briquet A., Lechanteur C., Detry O., Beguin Y., Jouret F. (2020). Mesenchymal Stromal Cells in Solid Organ Transplantation. Transplantation.

[31] Zingraf G., Derkowski D.M., Pizarro Y.I., Woodard A.U.S. (2023). Organ Transplantation System—Its History, Present, and Future. Nephrol. Nurs. J..

[32] Jadlowiec C.C., Taner T. (2016). Liver transplantation: Current status and challenges. World J. Gastroenterol..

[33] Capuzzimati M., Hough O., Liu M. (2022). Cell death and ischemia-reperfusion injury in lung transplantation. J. Heart Lung Transplant..

[34] Poudel S., Gupta S., Saigal S. (2024). Basics and Art of Immunosuppression in Liver Transplantation. J. Clin. Exp. Hepatol..

[35] Uluer M.C., Brazio P.S., Woodall J.D., Nam A.J., Bartlett S.T., Barth R.N. (2016). Vascularized Composite Allotransplantation: Medical Complications. Curr. Transplant. Rep..

[36] Morelon E., Petruzzo P., Kanitakis J. (2018). Chronic rejection in vascularized composite allotransplantation. Curr. Opin. Organ Transplant..

[37] Amin K.R., Fildes J.E. (2024). The contribution of the donor vascularised hand and face allograft in transplant rejection: An immunological perspective. Transpl. Immunol..

[38] Iafolla M.A., Tay J., Allan D.S. (2014). Transplantation of umbilical cord blood-derived cells for novel indications in regenerative therapy or immune modulation: A scoping review of clinical studies. Biol. Blood Marrow Transplant..

[39] Damien P., Allan D.S. (2015). Regenerative Therapy and Immune Modulation Using Umbilical Cord Blood-Derived Cells. Biol. Blood Marrow Transplant..

[40] Rizk M., Aziz J., Shorr R., Allan D.S. (2017). Cell-Based Therapy Using Umbilical Cord Blood for Novel Indications in Regenerative Therapy and Immune Modulation: An Updated Systematic Scoping Review of the Literature. Biol. Blood Marrow Transplant..

[41] Liao Y., Geyer M.B., Yang A.J., Cairo M.S. (2011). Cord blood transplantation and stem cell regenerative potential. Exp. Hematol..

[42] Um S., Ha J., Choi S.J., Oh W., Jin H.J. (2020). Prospects for the therapeutic development of umbilical cord blood-derived mesenchymal stem cells. World J. Stem Cells.

[43] Van S.Y., Noh Y.K., Kim S.W., Oh Y.M., Kim I.H., Park K. (2019). Human umbilical cord blood mesenchymal stem cells expansion via human fibroblast-derived matrix and their potentials toward regenerative application. Cell Tissue Res..

[44] Broxmeyer H.E., Hangoc G., Cooper S., Ribeiro R.C., Graves V., Yoder M., Wagner J., Vadhan-Raj S., Benninger L., Rubinstein P. (1992). Growth characteristics and expansion of human umbilical cord blood and estimation of its potential for transplantation in adults. Proc. Natl. Acad. Sci. USA.

[45] Nagamura-Inoue T., Nagamura F. (2023). Umbilical cord blood and cord tissue banking as somatic stem cell resources to support medical cell modalities. Inflamm. Regen..

[46] Wang J., Metheny L. (2023). Umbilical cord blood derived cellular therapy: Advances in clinical development. Front. Oncol..

[47] Wang M., Yang Y., Yang D., Luo F., Liang W., Guo S., Xu J. (2009). The immunomodulatory activity of human umbilical cord blood-derived mesenchymal stem cells in vitro. Immunology.

[48] Allan D.S. (2020). Using umbilical cord blood for regenerative therapy: Proof or promise?. Stem Cells.

[49] Dumont-Lagacé M., Feghaly A., Meunier M.C., Finney M., Van’t Hof W., Masson Frenet E., Sauvageau G., Cohen S. (2022). UM171 Expansion of Cord Blood Improves Donor Availability and HLA Matching For All Patients, Including Minorities. Transplant. Cell Ther..

[50] Wall D.A., Chan K.W. (2008). Selection of cord blood unit(s) for transplantation. Bone Marrow Transplant..

[51] Sica R.A., Terzioglu M.K., Mahmud D., Mahmud N. (2020). Mechanistic Basis of ex Vivo Umbilical Cord Blood Stem Progenitor Cell Expansion. Stem Cell Rev. Rep..

[52] Ren Y., Cui Y., Tan Y., Xu Z., Wang H. (2023). Expansion strategies for umbilical cord blood haematopoietic stem cells in vitro. Vox Sang..

[53] Chivu M., Diaconu C.C., Bleotu C., Alexiu I., Brasoveanu L., Cernescu C. (2004). The comparison of different protocols for expansion of umbilical-cord blood hematopoietic stem cells. J. Cell. Mol. Med..

[54] Roura S., Pujal J.M., Gálvez-Montón C., Bayes-Genis A. (2015). The role and potential of umbilical cord blood in an era of new therapies: A review. Stem Cell Res. Ther..

[55] Mousavi S.H., Zarrabi M., Abroun S., Ahmadipanah M., Abbaspanah B. (2019). Umbilical cord blood quality and quantity: Collection up to transplantation. Asian J. Transfus. Sci..

[56] Devi S., Bongale A.M., Tefera M.A., Dixit P., Bhanap P. (2023). Fresh Umbilical Cord Blood-A Source of Multipotent Stem Cells, Collection, Banking, Cryopreservation, and Ethical Concerns. Life.

[57] Scaradavou A. (2024). Cord Blood Stem Cells Do Not “Age”-Under Proper Banking Conditions. Stem Cells Transl. Med..

[58] Almici C., Carlo-Stella C., Wagner J.E., Mangoni L., Garau D., Re A., Giachetti R., Cesana C., Rizzoli V. (1997). Clonogenic capacity and ex vivo expansion potential of umbilical cord blood progenitor cells are not impaired by cryopreservation. Bone Marrow Transplant..

[59] Moezzi L., Pourfathollah A.A., Alimoghaddam K., Soleimani M., Ardjmand A.R. (2005). The effect of cryopreservation on clonogenic capacity and in vitro expansion potential of umbilical cord blood progenitor cells. Transplant. Proc..

[60] M-Reboredo N., Díaz A., Castro A., Villaescusa R.G. (2000). Collection, processing and cryopreservation of umbilical cord blood for unrelated transplantation. Bone Marrow Transplant..

[61] Yasui K., Ogawa Y., Saino O., Akamatsu R., Fuchizaki A., Irie Y., Nabetani M., Tanaka M., Takihara Y., Taguchi A. (2024). X-irradiated umbilical cord blood cells retain their regenerative effect in experimental stroke. Sci. Rep..

[62] El-Naseery N.I., Elewa Y.H.A., El-Behery E.I., Dessouky A.A. (2023). Human umbilical cord blood-derived mesenchymal stem cells restored hematopoiesis by improving radiation induced bone marrow niche remodeling in rats. Ann. Anat..

[63] Starzl T.E., Demetris A.J., Trucco M., Ricordi C., Ildstad S., Terasaki P.I., Murase N., Kendall R.S., Kocova M., Rudert W.A. (1993). Chimerism after liver transplantation for type IV glycogen storage disease and type 1 Gaucher’s disease. N. Engl. J. Med..

[64] Starzl T.E., Demetris A.J., Murase N., Ildstad S., Ricordi C., Trucco M. (1992). Cell migration, chimerism, and graft acceptance. Lancet.

[65] Starzl T.E., Murase N., Demetris A.J., Giorda R., Valdivia L., Trucco M. (1993). Drug development and testing in relation to cell migration and chimerism. Transplant. Proc..

[66] Starzl T.E., Demetris A.J., Trucco M., Zeevi A., Ramos H., Terasaki P., Rudert W.A., Kocova M., Ricordi C., Ildstad S. (1993). Chimerism and donor-specific nonreactivity 27 to 29 years after kidney allotransplantation. Transplantation.

[67] Starzl T.E., Demetris A.J., Trucco M., Murase N., Ricordi C., Ildstad S., Ramos H., Todo S., Tzakis A., Fung J.J. (1993). Cell migration and chimerism after whole-organ transplantation: The basis of graft acceptance. Hepatology.

[68] Lovasova V., Bem R., Chlupac J., Dubsky M., Husakova J., Nemcova A., Fronek J. (2022). Animal experimental models of ischemic wounds—A review of literature. Wound Repair. Regen..

[69] Gutt C.N., Riemer V., Brier C., Berguer R., Paolucci V. (1998). Standardized technique of laparoscopic surgery in the rat. Dig. Surg..

[70] Siemionow M., Cwykiel J., Heydemann A., Garcia J., Marchese E., Siemionow K., Szilagyi E. (2018). Dystrophin Expressing Chimeric (DEC) Human Cells Provide a Potential Therapy for Duchenne Muscular Dystrophy. Stem Cell Rev. Rep..

[71] Heydemann A., Bieganski G., Wachowiak J., Czarnota J., Niezgoda A., Siemionow K., Ziemiecka A., Sikorska M.H., Bozyk K., Tullius S.G. (2023). Dystrophin Expressing Chimeric (DEC) Cell Therapy for Duchenne Muscular Dystrophy: A First-in-Human Study with Minimum 6 Months Follow-up. Stem Cell Rev. Rep..

[72] Siemionow M., Biegański G., Niezgoda A., Wachowiak J., Czarnota J., Siemionow K., Ziemiecka A., Sikorska M.H., Bożyk K., Heydemann A. (2023). Safety and Efficacy of DT-DEC01 Therapy in Duchenne Muscular Dystrophy Patients: A 12—Month Follow-Up Study After Systemic Intraosseous Administration. Stem Cell Rev. Rep..

[73] Kiritsi D., Dieter K., Niebergall-Roth E., Fluhr S., Daniele C., Esterlechner J., Sadeghi S., Ballikaya S., Erdinger L., Schauer F. (2021). Clinical trial of ABCB5+ mesenchymal stem cells for recessive dystrophic epidermolysis bullosa. J. Clin. Investig..

[74] Dick J.E., Lapidot T., Pflumio F. (1991). Transplantation of normal and leukemic human bone marrow into immune-deficient mice: Development of animal models for human hematopoiesis. Immunol. Rev..

[75] Lapidot T., Fajerman Y., Kollet O. (1997). Immune-deficient SCID and NOD/SCID mice models as functional assays for studying normal and malignant human hematopoiesis. J. Mol. Med..

[76] Wuttisarnwattana P., Eid S., Gargesha M., Cooke K.R., Wilson D.L. (2020). Cryo-imaging of Stem Cell Biodistribution in Mouse Model of Graft-Versus-Host-Disease. Ann. Biomed. Eng..

[77] Zhou D.H., Huang S.L., Huang K., Wu Y.F., Bao R., Wei J., Zhang X.C., Li Y. (2005). Mesenchymal stem cells from human cord blood promote engraftment of human umbilical cord blood-derived CD34+ cells in NOD/SCID mice. Zhonghua Xue Ye Xue Za Zhi.

[78] Hao M., Qi P.J., Li G., Meng H.X., Xu Y., Li C.H., Wang Y.F., Qiu L.G. (2010). Effect of human umbilical cord mesenchymal stem cells on the CD34+ cells transplantation in NOD/SCID mice. Zhongguo Yi Xue Ke Xue Yuan Xue Bao.

[79] Huang Z., Xiao Y., Chen X., Li H., Gao J., Wei W., Zhang X., Feng X. (2021). Cotransplantation of Umbilical Cord Mesenchymal Stem Cells Promotes the Engraftment of Umbilical Cord Blood Stem Cells in Iron Overload NOD/SCID Mice. Transplant. Cell Ther..

[80] Gao J.T., Lu S.H., Li Y.H., Yang Z., Xu J., Zheng Y.Z. (2008). The observation of engraftment of human umbilical cord blood-derived hematopoietic stem/progenitor cells in xenotransplanted NOD/SCID mouse model by intra-bone marrow injection. Zhonghua Xue Ye Xue Za Zhi.

[81] Noort W.A., Kruisselbrink A.B., in’t Anker P.S., Kruger M., van Bezooijen R.L., de Paus R.A., Heemskerk M.H., Löwik C.W., Falkenburg J.H., Willemze R. (2002). Mesenchymal stem cells promote engraftment of human umbilical cord blood-derived CD34(+) cells in NOD/SCID mice. Exp. Hematol..

[82] Fibbe W.E., Noort W.A., Schipper F., Willemze R. (2001). Ex vivo expansion and engraftment potential of cord blood-derived CD34+ cells in NOD/SCID mice. Ann. N. Y. Acad. Sci..

[83] Gao J., Li Y., Lu S., Wang M., Yang Z., Yan X., Zheng Y. (2009). Enhanced in vivo motility of human umbilical cord blood hematopoietic stem/progenitor cells introduced via intra-bone marrow injection into xenotransplanted NOD/SCID mouse. Exp. Hematol..

[84] Zhou Q., Facciponte J., Jin M., Shen Q., Lin Q. (2014). Humanized NOD-SCID IL2rg–/– mice as a preclinical model for cancer research and its potential use for individualized cancer therapies. Cancer Lett..

[85] Pearson T., Greiner D.L., Shultz L.D. (2008). Humanized SCID mouse models for biomedical research. Curr. Top. Microbiol. Immunol..

[86] Chen J., Liao S., Xiao Z., Pan Q., Wang X., Shen K., Wang S., Yang L., Guo F., Liu H.F. (2022). The development and improvement of immunodeficient mice and humanized immune system mouse models. Front. Immunol..

[87] Gao L., Wang J.M., Xie L.N., Zhou H., Qiu H.Y. (2008). Establishment of an xenogeneic acute graft-versus-host disease model in NOD/SCID mice by engraftment of G-CSF mobilized human mononuclear cells. Zhonghua Xue Ye Xue Za Zhi.

[88] Massollo M., Podestà M., Marini C., Morbelli S., Cassanelli C., Pinto V., Ubezio G., Curti G., Uccelli A., Frassoni F. (2010). Contact with the bone marrow microenvironment readdresses the fate of transplanted hematopoietic stem cells. Exp. Hematol..

[89] Fischer U.M., Harting M.T., Jimenez F., Monzon-Posadas W.O., Xue H., Savitz S.I., Laine G.A., Cox C.S. (2009). Pulmonary passage is a major obstacle for intravenous stem cell delivery: The pulmonary first-pass effect. Stem Cells Dev..

[90] Ferrini E., Stellari F.F., Franceschi V., Macchi F., Russo L., Murgia A., Grisendi G., Villetti G., Dominici M., Donofrio G. (2021). Persistency of Mesenchymal Stromal/Stem Cells in Lungs. Front. Cell Dev. Biol..

[91] Tappenbeck N., Schröder H.M., Niebergall-Roth E., Hassinger F., Dehio U., Dieter K., Kraft K., Kerstan A., Esterlechner J., Frank N.Y. (2019). In vivo safety profile and biodistribution of GMP-manufactured human skin-derived ABCB5-positive mesenchymal stromal cells for use in clinical trials. Cytotherapy.

[92] Kean T.J., Lin P., Caplan A.I., Dennis J.E. (2013). MSCs: Delivery Routes and Engraftment, Cell-Targeting Strategies, and Immune Modulation. Stem Cells Int..

[93] Nystedt J., Anderson H., Tikkanen J., Pietilä M., Hirvonen T., Takalo R., Heiskanen A., Satomaa T., Natunen S., Lehtonen S. (2013). Cell surface structures influence lung clearance rate of systemically infused mesenchymal stromal cells. Stem Cells.

[94] Su W., Zhou M., Zheng Y., Fan Y., Wang L., Han Z., Kong D., Zhao R.C., Wu J.C., Xiang R. (2011). Bioluminescence reporter gene imaging characterize human embryonic stem cell-derived teratoma formation. J. Cell. Biochem..

[95] Pomper M.G., Hammond H., Yu X., Ye Z., Foss C.A., Lin D.D., Fox J.J., Cheng L. (2009). Serial imaging of human embryonic stem-cell engraftment and teratoma formation in live mouse models. Cell Res..

[96] Cao F., Li Z., Lee A., Liu Z., Chen K., Wang H., Cai W., Chen X., Wu J.C. (2009). Noninvasive de novo imaging of human embryonic stem cell-derived teratoma formation. Cancer Res..

[97] Medvedev S.P., Shevchenko A.I., Zakian S.M. (2010). Induced Pluripotent Stem Cells: Problems and Advantages when Applying them in Regenerative Medicine. Acta Naturae.

[98] Okita K., Ichisaka T., Yamanaka S. (2007). Generation of germline-competent induced pluripotent stem cells. Nature.

[99] Xia X., Li H., Satheesan S., Zhou J., Rossi J.J. (2019). Humanized NOD/SCID/IL2rγnull (hu-NSG) Mouse Model for HIV Replication and Latency Studies. J. Vis. Exp..

[100] Ma L.J., Hu X.X., Zhou H., Gao L., Xie L.N., Qiu H.Y., Wang J.M. (2008). Study on hematopoiesis reconstitution by co-transplant of human bone marrow mesenchymal stem cells and umbilical cord blood CD34(+) cells at different ratios in NOD/SCID mice. Zhonghua Xue Ye Xue Za Zhi.

